# Systematic analyses of the role of prognostic and immunological EIF3A, a reader protein, in clear cell renal cell carcinoma

**DOI:** 10.1186/s12935-021-02364-2

**Published:** 2021-12-19

**Authors:** Yi Zhang, Xiaoliang Hua, Haoqiang Shi, Li Zhang, Haibing Xiao, Chaozhao Liang

**Affiliations:** 1 Department of Urology, The First Affiliated Hospital of Anhui Medical University, Anhui Medical University, Hefei, Anhui China; 2 Institute of Urology, Anhui Medical University, Hefei, Anhui China; 3 Anhui Province Key Laboratory of Genitourinary Diseases, Anhui Medical University, Hefei, Anhui China

**Keywords:** EIF3A, Clear cell renal cell carcinoma, N6-methyladenosine (m6A) RNA methylation, Overall survival, Tumour-immune infiltration

## Abstract

**Background:**

Eukaryotic initiation factor 3a (EIF3A), a “reader” protein for RNA methylation, has been found to be involved in promoting tumorigenesis in a variety of cancers. The impact of EIF3A in clear cell renal cell carcinoma (ccRCC) has yet to be reported. This study aimed to identify the prognostic value of EIF3A in ccRCC and investigate the relationship between EIF3A expression and immune infiltration.

**Methods:**

We collected 29 m6A-related mRNA data and clinicopathological parameters from The Cancer Genome Atlas (TCGA) database. Logistic regression analyses were used to analyse the correlation between EIF3A expression and clinical characteristics. Immunohistochemistry (IHC) was applied to examine EIF3A levels in normal and ccRCC tissues. Univariate and multivariate analyses were conducted to recognize independent factors associated with overall survival (OS) and disease-free survival (DFS). The nomogram aimed to predict the 1-, 3- and 5-year survival probabilities. Gene set enrichment analysis (GSEA) was carried out to determine the potential functions and related signalling pathways of EIF3A expression. To investigate EIF3A of coexpressed genes, we used LinkedOmics, and the results were subjected to enrichment analysis. Simultaneously, LinkedOmics and STRING datasets were used to identify EIF3A coexpressed genes that were visualized via Cytoscape. Finally, we evaluated whether EIF3A expression correlated with the infiltration of immune cells and the expression of marker genes in ccRCC by Tumour Immune Estimation Resource (TIMER) and Gene Expression Profiling Interactive Analysis (GEPIA).

**Result:**

EIF3A expression was significantly different between ccRCC tissues and normal tissues. EIF3A expression was correlated with poor prognostic clinicopathological factors, and K–M analyses revealed that low EIF3A expression was correlated with a poor prognosis. The results of univariate and multivariate analyses proved that EIF3A was a prognostic factor in ccRCC patients. GSEA results indicated that EIF3A high expression was enriched in the renal cell carcinoma pathway. EIF3A expression was significantly positively correlated with B cells, CD8 + T cells, CD4 + T cells, neutrophils, macrophages, and dendritic cells. Furthermore, EIF3A expression was associated with most marker genes of immune cells.

**Conclusions:**

EIF3A could serve as a potential biomarker for prognostic and diagnostic stratification of ccRCC and is related to immune cell infiltrates.

**Supplementary Information:**

The online version contains supplementary material available at 10.1186/s12935-021-02364-2.

## Background

The morbidity of renal cell carcinoma (RCC) is second only to prostate cancer and bladder cancer [1] among urinary system malignancies, and it has a high mortality and recurrence rate. It is estimated that RCC is the seventh most common cancer in men and the ninth in women, with 48,780 newly diagnosed cases and 27,300 new cases of RCC-related mortality in the United States [2]. Overall, the worldwide incidence has increased by 2% per year during the last 2 decades [3]. Clear cell RCC is the most frequent renal cell carcinoma (ccRCC), accounting for approximately 80–90% of all kidney cancers [4]. Surgery is still the most curative treatment for localized RCC, and there are therapeutic approaches as alternatives to surgery, including embolization, ablative therapies, targeted therapies, immunotherapy and adjuvant therapy [5]. However, the treatment results are not satisfactory, and the mortality rates are stubbornly high. Therefore, the identification of biomarkers for ccRCC can improve its prognostic systems, which urgently needs to be addressed.

N6-methyladenosine (m6A), an epigenetic modification, is the most prevalent methylation in eukaryotic mRNAs and was first discovered in 1974 [6, 7]. With the rapid development of high-throughput sequencing technology and further research, m6A was found to exist in various types of RNAs [8]. Subsequently, m6A was discovered to be involved in various aspects of RNA metabolism, including pre-mRNA splicing, 3′ end processing, nuclear export, translation regulation, mRNA decay, and noncoding RNA (ncRNA) processing [9]. Recent reports have shown that it is closely related to the regulation of gene expression at the posttranscriptional level, biological development, and human diseases, especially tumorigenesis and progression [10].

Meanwhile, m6A modification marks a new direction for oncotherapy [11], and the differential expression of m6A regulators in different types of tumours can significantly affect the prognosis of patients [12]. The regulation of m6A modification is a dynamic and reversible process. M6A methyltransferases are called “Writers”, such as methyltransferase-like protein 3/14 (METTL3/14), wt1-associated protein (WTAP), and vir-likem6A methyltransferase associated protein (VIRMA). Fat mass and obesity-associated protein (FTO) and alkylation repair homologue 5 (ALKBH5) can remove the m6A mark and induce demethylation, called Erasers”. All kinds of Readers can bind to the m6A modification site in RNA and thereby have different effects on targeted mRNAs, including the YT521-B homology (YTH) domain family, heterogeneous nuclear ribonucleoproteins (HNRNPs) and insulin-like growth factor 2 mRNA-binding proteins (IGF2BPs, including IGF2BP1/2/3). Previous studies have mentioned the influence of the expression and impact of m6A-related genes in ccRCC [13, 14]. We collected 29 m6A-related genes, and the EIF3A gene was selected to study its role in ccRCC.

EIF3A, a “writer”, is the largest subunit of EIF3, which is a critical factor in translation initiation. EIF3A can bind with the 5′UTR to promote the translation of cap-independent mRNAs [15]. Evidence suggests that EIF3A is a proto-oncogene involved in tumorigenesis and metastasis in the lung [16], colon [17], stomach [18] and urinary bladder [19]. The expression of EIF3A can influence cancer cell growth, and the malignant phenotype of cancer cells can be reversed by knocking down EIF3A [20].

A previous study used whole exome sequencing to identify mortality-related somatic mutations in ccRCC, and subsequent validation of the results showed that only SIPA1L2 and EIF3A were associated with the ccRCC prognosis out of 138 prioritized genes, which can better evaluate the impact on ccRCC patient mortality. [21]. EIF3A can affect the resistance to some anticancer drugs, whether knocked down or overexpressed [22]. In general, high expression of EIF3A being associated with better survival is not consistent with what we recognize as a proto-oncogene, and its mechanisms of action in cancer tumorigenesis and prognosis remain unknown.

Hence, we investigated the correlation between EIF3A expression and clinical and pathological characteristics and the prognostic value of EIF3A. Gene set enrichment analysis (GSEA) and GEPIA were undertaken. Furthermore, the Linkedomics and STRING datasets were utilized to analyse coexpression and visualized via Cytoscape. Finally, the relationship of EIF3A expression and infiltration of immune cells and marker gene expression in ccRCC was researched by Tumour Immune Estimation Resource (TIMER).

## Materials and methods

### Dataset acquisition

The RNA-seq transcriptome data and clinicopathological information from 539 ccRCC samples and 72 normal samples were retrieved from the TCGA database (https://portal.gdc.cancer.gov/). A total of 29 m6A-related genes were selected (METTL3, METTL14, METTL13, WTAP, RBM15, RBM15B, ZC3H13, NSun2, MTCH2, CBLL1, ALKBH3, FTO, ALKBH5, YTHDF1, YTHDF2, YTHDF3, YTHDC1, YTHDC2, HNRNPA2B1, HNRNPC, HNRNPG, LRPPRC, FMR1, IGF2BP1, IGF2BP2, IGF2BP3, EIF3A, NKAP, and KIAA1429). The RNA-seq data underwent normalization.

### Analyses of the association between EIF3A expression and clinical, pathological characteristics

Logistic regression analyses and independent sample t-tests were utilized to analyse the correlation between EIF3A expression and the clinical and pathological characteristics of ccRCC. According to the median EIF3A expression, patients were divided into a high-expression group and a low-expression group. Then, we assessed the survival difference between the groups by the Kaplan–Meier (K–M) method and the log-rank test. Based on the receiver operating characteristic curves (ROCs) and the area under the curve (AUC), we evaluated the specificity and sensitivity of EIF3A.

### Immunohistochemistry (IHC) and colon, pancreatic cancer tissue and human ccRCC tissue arrays

Human ccRCC tumour tissue arrays were purchased from Shanghai Superchip (Biochip Lot No. XT15-050, CGt No. HKidE180Su02, website address: http://www.superchip.com.cn/biology/tissue.html, Shanghai, China), and 150 ccRCC and 30 corresponding nontumour tissues were purchased from BioChip (Shanghai, China). Pancreatic and colon cancers and their adjacent tissues were paraffin embedded tissues, and we sectioned them (The study protocol was approved by the ethics committee of The First Affiliated Hospital of An Hui Medical University and a written informed consent was obtained from all participants involved in this study). The tissue array sections and paraffin embedded tissues were dehydrated and subjected to peroxidase blocking by H_2_O_2_. Then, heat-mediated antigen retrieval was performed using citrate buffer. After treating the tissue arrays with 5% BSA for 20 min at room temperature, anti-EIF3A antibody was added and incubated at room temperature for 1 h. After washing with PBS, the sections were subjected to indirect immunohistochemistry using HRP-labelled goat antirabbit IgG (Thermo Scientific). Next, DAB substrate (ab80437, Abcam) was added and incubated for 1–10 min. The tissue array sections were counterstained with haematoxylin. Images were taken with a microscope. The mean proportion of stained cells per specimen was determined semiquantitatively and scored as follows: 0 for staining 0–1%, 1 for 1–25%, 2 for 26–50%, 3 for 51–75%, and 4 for > 75% of the examined cells. The staining intensity was graded as follows: 0, negative staining; 1, weak staining; 2, moderate staining; and 3, strong staining. The histological score (H-score) for each specimen was computed by the formula: H-score = Proportion score × Intensity score. Overall scores of < 6 and ≥ 6 were defined as negative and positive, respectively [23].

Deparaffinize and rehydrate: incubate sections in 2 changes of xylene, 15 min each. Dehydrate in 2 changes of pure ethanol for 5 min, followed by dehydrate in gradient ethanol of 85% and 75% ethanol, respectively, 5 min each. Wash in distilled water. Antigen retrieval: immerse the slides in EDTA antigen retrieval buffer (pH 8.0) and maintain at a sub-boiling temperature for 8 min, standing for 8 min and then followed by another sub-boiling temperature for 7 min. Be sure to prevent buffer solution evaporate. Let air cooling. Wash three times with PBS (pH 7.4) in a Rocker device, 5 min each. Use the right antigen retrieval buffer and heat extent according to tissue characteristics. Circle and Serum blocking: eliminate obvious liquid, mark the objective tissue with liquid blocker pen. Add 3% BSA to cover the marked tissue to block non-specific binding for 30 min. Cover objective area with 10% donkey serum (for the case of primary antibody originated from goat) or 3% BSA (for the case of primary antibody originated from others). Primary antibody: throw away the blocking solution slightly. Incubate slides with primary antibody (diluted with PBS appropriately) overnight at 4 ℃, placed in a wet box containing a little water. Secondary antibody: wash slides three times with PBS (pH 7.4) in a Rocker device, 5 min each. Then throw away liquid slightly. Cover objective tissue with secondary antibody (appropriately respond to primary antibody in species), incubate at room temperature for 50 min in dark condition. DAPI counterstain in nucleus: wash three times with PBS (pH 7.4) in a Rocker device, 5 min each. Then incubate with DAPI solution at room temperature for 10 min, kept in dark place. Spontaneous fluorescence quenching: wash three times with PBS (pH 7.4) in a Rocker device, 5 min each. Add spontaneous fluorescence quenching reagent to incubate for 5 min. Wash in running tap water for 10 min. Throw away liquid slightly, then cover slip with anti-fade mounting medium. Microscopy detection and collect images by Fluorescent Microscopy. DAPI glows blue by UV excitation wavelength 330–380 nm and emission wavelength 420 nm; FITC glows green by excitation wavelength 465–495 nm and emission wavelength 515–555 nm; CY3 glows red by excitation wavelength 510–560 nm and emission wavelength 590 nm.

### Univariate and multivariate cox hazard regression analyses

The independent prognostic factors were identified by univariate and multivariate Cox hazard regression, and ROC curves and AUC values of these eight factors were calculated, including EIF3A expression level, grade, T stage, N stage, M stage, age and sex.

### Nomogram predict survival probabilities and risk score

To predict 1-year, 3-year, and 5-year survival rates, we carried out visualization of the correlation between OS and various factors through the R “rms” package. The risk score (RS) was estimated by using the formula: Risk score = coefficient1 × EIF3A + coefficient N × clinical characteristics N. By means of the Kaplan–Meier (K–M) method, the log-rank test and ROC analyses, we determined whether the survival differences between two groups based on the median of the risk score were significant.

### Gene set enrichment analysis (GSEA)

GSEA is a computational method that compares the concordant differences between two groups (high expression and low expression) [24]. In the present study, a hallmark gene set was used to explore the potential mechanism and discover significant critical biological pathways of EIF3A expression in ccRCC. In general, it was considered to be significant when gene sets had a false discovery rate (FDR) < 0.25, absolute value of the normalized enrichment score (NES) ≥ 1.0, and normalized P < 0.05.

### Analyses of coexpressed genes

We used the LinkedOmics database to screen out genes that were coexpressed with EIF3A in ccRCC by Pearson’s correlation, and the results are presented as heatmaps and volcano plots [25]. To undertake functional annotations for coexpressed genes, the Gene Ontology (GO) database and KEGG database were used in Metascape, and the results are shown as bubble charts. Then, by means of the STRING database, we established the potential protein–protein interactions (PPIs) of coexpressed genes. PPI pairs were extracted with a minimum interaction score of 0.4, and the PPI network was visualized by Cytoscape 3.7.2. CytoHubba Plugin was used to identify the top 10 core genes in the gene interaction network and PPI network according to the degree score of each gene node.

### Immune cells infiltration

The relationship between EIF3A expression in ccRCC and the infiltration of immune cells, including B cells, CD4 + T cells, CD8 + T cells, macrophages, neutrophils, and dendritic cells (DCs), and tumour purity was analysed by using the Timer “Gene” module. In addition, the correlation between the expression of differential MMUNE cells and marker genes was analysed by correlation modules.

### Statistical analysis

All statistics and data were analysed by using SPSS 23.0 (IBM, Chicago, USA), R 4.05 (https://www.rproject.org/) and GraphPad Prism 8.0 (San Diego, CA, USA). The correlation between two different genes was analysed by the Pearson correlation method. To assess the associations between clinicopathological parameters and EIF3A, we used the chi-square test and logistic regression. We evaluated the diagnostic efficacy of EIF3A expression and RS by using Kaplan–Meier plotter and the log-rank test. Cox regression analysis was used to evaluate factors associated with overall survival (OS) and disease-free survival (DFS). The R statistical packages were used to draw the nomogram. All statistical results with P < 0.05 were statistically significant.

## Results

### mRNA expression level

**Fig. 1:**
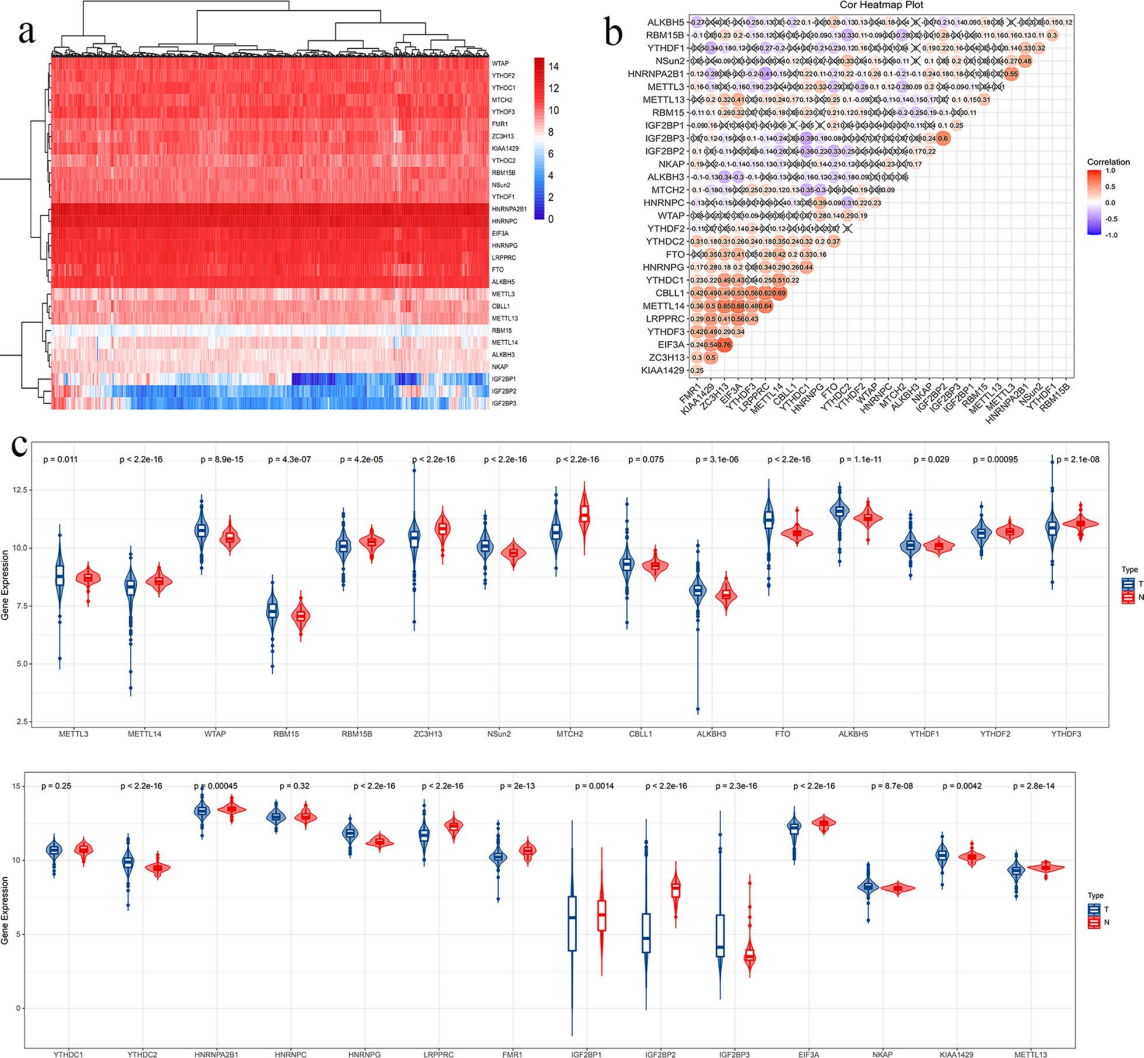
The expression of m6A-related genes in ccRCC; **a** Heatmaps of 29 m6a RNA-related genes expression levels in ccRCC and normal tissues; **b** Pearson correlations of these 29 differentially expressed m6A related genes; **c** Vioplots of the 29 differentially expressed m6A related genes in ccRCC and normal tissues

First, we analysed the expression levels of a total of 29 m6A-related genes. The results are shown as heatmaps in Fig. 1a. Figure 1b is a cor-heatmap that indicated the interaction of these genes by correlation analysis. The results indicate that EIF3A and ZC3H13 are the strongest relevant genes (Pearson’s r = 0.78). Compared to normal tissue, METTL3, WTAP, RBM15, HNRNPG, NKAP, KIAA1429, NSun2, ALKBH3, FTO, ALKBH5, YTHDF1, IGF2BP3 and YTHDC2 were down regulated (p < 0.05), while METTL14, RBM15B, ZC3H13, MTCH2, YTHDF2, YTHDF3, HNRNPA2B1, LRPPRC, FMR1, IGF2BP1, IGF2BP2, EIF3A, and METTL13 had relatively high expression (p < 0.05). There was no significant difference between HNRNPC, YTHDC1 and CBLL1 (p > 0.05) (Fig. 1c).

### Associations with clinical characteristics and EIF3A

**Fig. 2:**
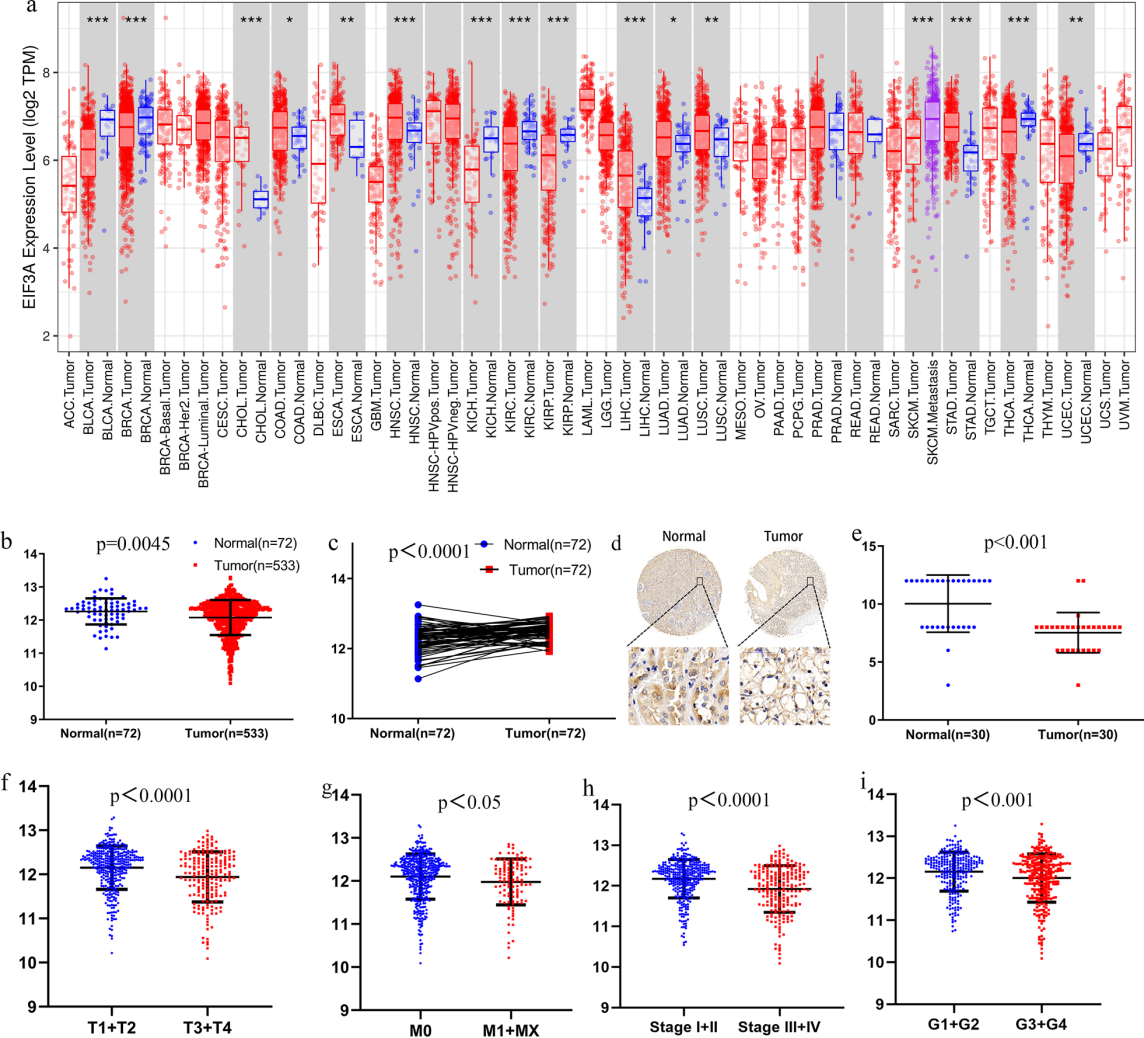
The expression level of EIF3A in tumor tissues and normal tissues; **a** The expression level of EIF3A in different types of tumor tissues and normal tissues in TIMER database (*P < 0.05, **P < 0.01, ***P < 0.001); **b** The expression level of EIF3A in 72 ccRCC tissues and 72 normal tissues; **c** The expression level of EIF3A in 533 ccRCC tissues and 72 normal tissues; **d** Immunohistochemical (IHC) analysis of EIF3A expression in ccRCC tissues and para-cancerous tissues; **e** Expression of EIF3A protein in 30 paired ccRCC tissues and normal renal tissues; The expression level of EIF3A correlated with various clinicopathological characteristics in ccRCC tissues: **f** T stages; **g** M stages; **h** Stage; **i** G stages

First, we used TIMER to assess the expression of EIF3A in different cancer types. We found that the expression of EIF3A was significantly higher than normal in CHOL, COAD, ESCA, HNSC, LIHC and STAD. Conversely, EIF3A expression was significantly lower than normal in BLCA, BRCA, KICH, KIRC, KIRP, LUAD, LUSC, THCA and UCEC (Fig. 2a). We analysed the relationship between clinicopathological parameters and EIF3A expression in ccRCC (Table 1). EIF3A had low expression in tumour tissues relative to normal tissues and paired ccRCC tissues (Fig. 2b), and the expression of EIF3A in ccRCC tissues was significantly lower than that in nontumour tissues (Fig. 2c). Then, we analysed the relationship between clinicopathological parameters and EIF3A expression in ccRCC by independent sample t-tests. Immunohistochemical analysis of EIF3A protein expression showed that EIF3A staining was weaker in ccRCC tissues than in normal kidney tissues (Fig. 2d). Meanwhile, we also detected the expression of EIF3A in other tissues, and found that it was low in para-pancreatic cancer tissues and high in para-colon cancer tissues (Figs. 1).Subsequently, EIF3A expression was detected in renal cancer cells and paracancerous tissues in our own paired samples of 30 patients, and the results showed that EIF3A was also highly expressed in paracancerous tissues compared to tumour tissues (Fig. 2e). The results showed that EIF3A expression was lower in high T stage (p < 0.0001; Fig. 2f), M stage (p < 0.05; Fig. 2g), pathologic stage (p < 0.0001; Fig. 2h), and G stage (p < 0.001; Fig. 2i). Moreover, the expression of EIF3A in both OS and DFS was substantially correlated with high N stage (OS, OR = 0.337) in ccRCC patients by univariate logistic regression analyses (Table 2). These results revealed that in ccRCC patients, low EIF3A expression tended to be associated with a more advanced grade and stage than high EIF3A expression.

**Table 1:** Correlation between EIF3A mRNA expression and clinicopathological parameters of ccRCC pantties (*)

Parameter	EIF3A mRNA expression			χ2	p value
	Low	High	Number		
Age					
< 61	124	140	264	1.804	0.179
≥ 61	142	127	269		
Gender					
Male	179	166	345	1.835	0.176
Female	86	102	188		
T stage					
T1 + T2	110	194	304	53.292	0.0001
T3 + T4	156	73	229		
N stage					
N0	124	115	239	0.677	0.411
N1 + NX	142	152	294		
M stage					
M0	200	222	422	5.118	0.024
M1 + MX	66	45	111		
G stage*					
G1 + G2	102	141	243	11.342	0.001
G3 + G4	161	123	284		
Stage					
I + II	137	188	325	20.021	0.0001
III + IV	129	79	208		

*There are 6 cases of GX in the original data that cannot be classified by G stage and are eliminated. The total number of cases is 527

**Table 2:** EIF3A expression associated with clinical pathological variables (logistic regression)

Risk factors	EIF3A mRNA expression			EIF3A mRNA expression		
	OS			DFS		
	HR	P-value	95% CI	OR	P-value	95% CI
Age	1.122	0.529	0.784–1.606	0.988	0.109	0.973–1.003
Grade	1.362	0.114	0.929–1.996	0.735	0.827	0.581–1.971
Stage	2.198	0.14	0.773–6.250	0.376	0.082	0.125–1.134
T	0.967	0.946	0.362–2.582	1.24	0.684	0.440–3.494
N	0.337	0.046	0.116–0.979	2.898	0.052	0.992–8.464
M	1.018	0.954	0.561–1.847	1.07	0.827	0.581–1.971
Gender	1.213	0.314	0.833–1.766	0.818	0.297	0.561–1.193

### EIF3A is an independent prognostic factor

**Fig. 3:**
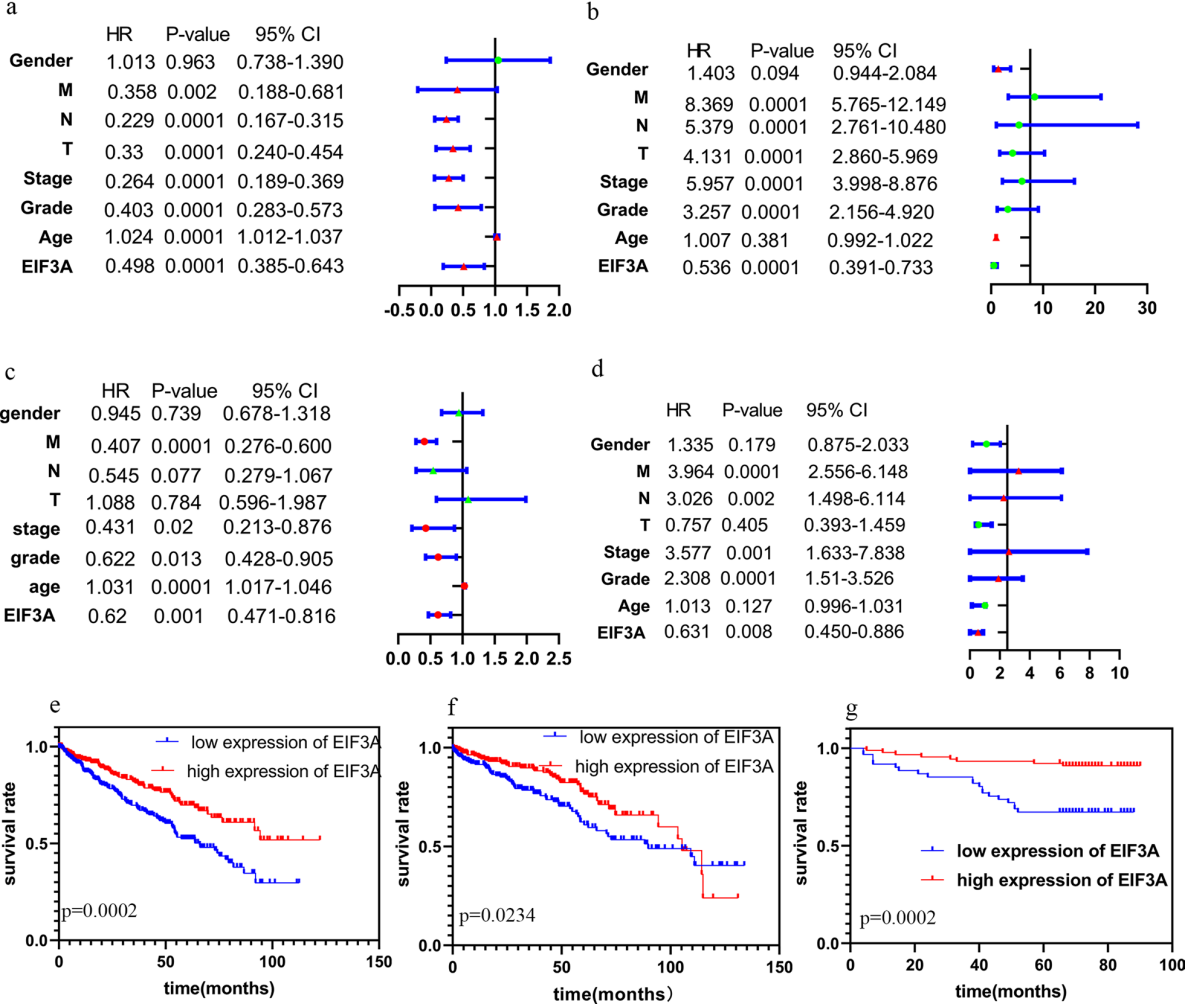
EIF3A as a independent prognostic factors and potential prognostic ability; **a** (OS), **b** (DFS) Univariate cox regression analyses; **c** (OS), **d** (DFS) multivariate cox regression analyses; **e**, **f** Kaplan–Meier survival curve shows EIF3A expression as OS and DFS in ccRCC (TCGA-ccRCC); **g** Kaplan–Meier survival curve of 150 patients with ccRCC

**Fig. 4:**
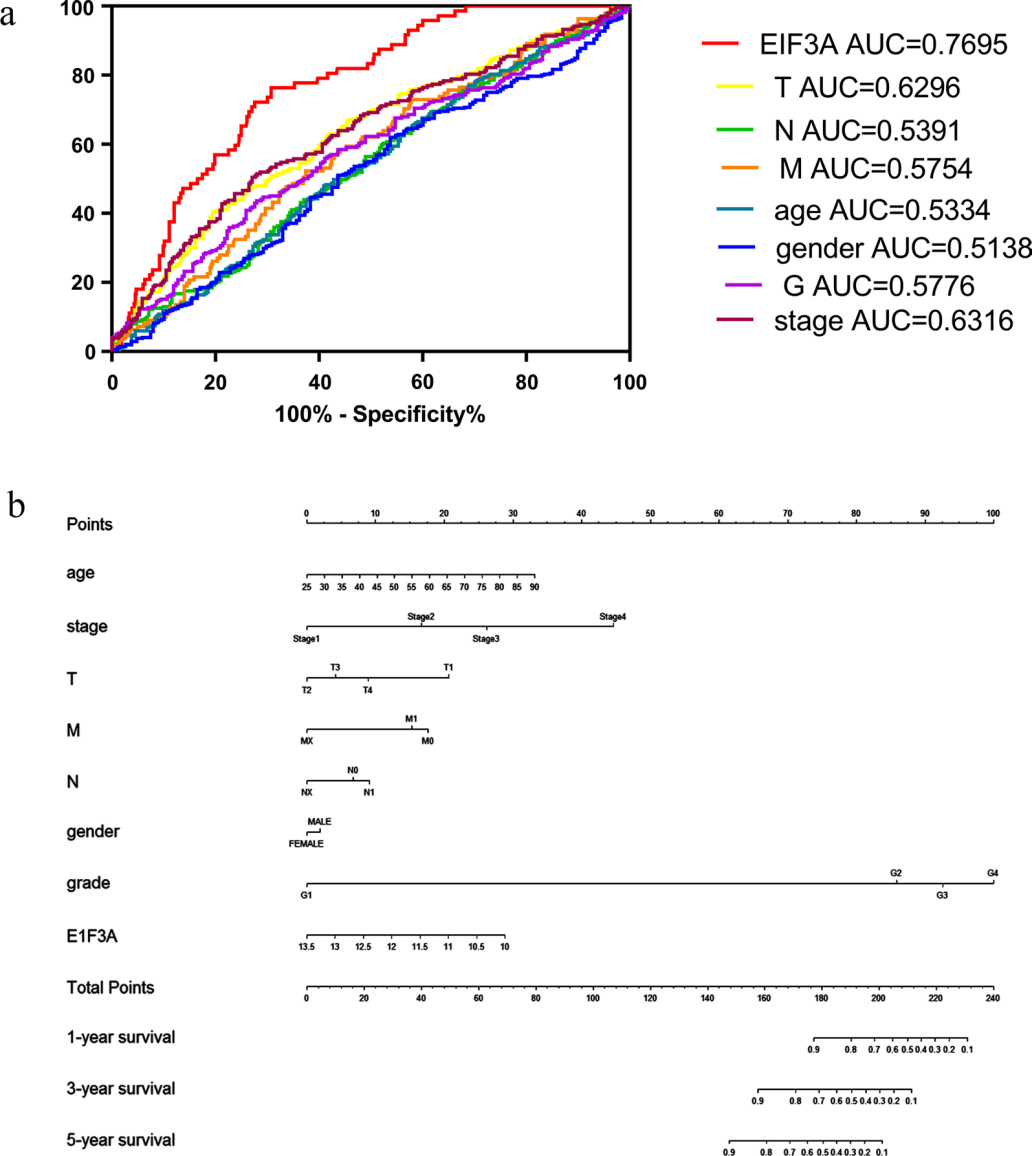
**a** MultiROC analysis of EIF3A expression and clinicopathological parameters of OS; **b** Established nomogram to predict the 1-, 3-, and 5-year survival probabilities

To investigate the association between EIF3A mRNA and OS or DFS in ccRCC patients, we used univariate and multivariate analyses. The univariate analysis results indicated that prognosis was not related to age or EIF3A expression for either OS (Table 3) or DFS (Table 3), and N stage was not related to DFS. The remaining factors were significantly associated with EIF3A expression in univariate analyses. By mean of the multivariate Cox analysis, we reached the conclusion that prognosis was related to the clinical stage (HR = 0.431, p = 0.02, 95% CI 0.213–0.876), grade (HR = 0.622, p = 0.013, 95% CI 0.428–0.905), M stage (HR = 0.407, p = 0.0001, 95% CI 0.276–0.600), age (HR = 1.031, p = 0.0001, 95% CI 1.017–1.046), and EIF3A expression (HR = 0.62, p = 0.001, 95% CI 0.471–0.816) in overall survival (Table 3). The results of the Cox analysis are shown in a forest plot (Fig. 3a–d). Kaplan–Meier analyses with log-rank tests were also carried out to demonstrate that the low-expression group had significantly shorter survival times than the high-expression group for both OS and DFS (Fig. 3e–f). A survival analysis of our own 150 ccRCC samples was performed to verify these conclusions (Fig. 3g). Furthermore, ROC curves showed that the predictive ability of EIF3A expression was favourable (AUC = 0.7695) (Fig. 4a).

**Table 3:** Univariate and multivariate analysis of EIF3A of overall and disease-free survival

Risk factors	Univariate analyses			Multivariate analyses			Univariate analyses			Multivariate analyses		
	OS						DFS					
	HR	P-value	95% CI	HR	P-value	95% CI	HR	P-value	95% CI	HR	P-value	95% CI
EIF3A	0.498	0.0001	0.385–0.643	0.62	0.001	0.471–0.816	0.536	0.0001	0.391–0.733	0.63	0.008	0.450–0.886
Age	1.024	0.0001	1.012–1.037	1.03	0.0001	1.017–1.046	1.007	0.381	0.992–1.022	1.01	0.127	0.996–1.031
Grade	0.403	0.0001	0.283–0.573	0.62	0.013	0.428–0.905	3.257	0.0001	2.156–4.920	2.31	0.0001	1.51–3.526
Stage	0.264	0.0001	0.189–0.369	0.43	0.02	0.213–0.876	5.957	0.0001	3.998–8.876	3.58	0.001	1.633–7.838
T	0.33	0.0001	0.240–0.454	1.09	0.784	0.596–1.987	4.131	0.0001	2.860–5.969	0.76	0.405	0.393–1.459
N	0.229	0.0001	0.167–0.315	0.55	0.077	0.279–1.067	5.379	0.0001	2.761–10.480	3.03	0.002	1.498–6.114
M	0.358	0.002	0.188–0.681	0.41	0.0001	0.276–0.600	8.369	0.0001	5.765–12.149	3.96	0.0001	2.556–6.148
Gender	1.013	0.963	0.738–1.390	0.95	0.739	0.678–1.318	1.403	0.094	0.944–2.084	1.34	0.179	0.875–2.033

### Construction of the nomogram and risk signature for internal validation

**Fig. 5:**
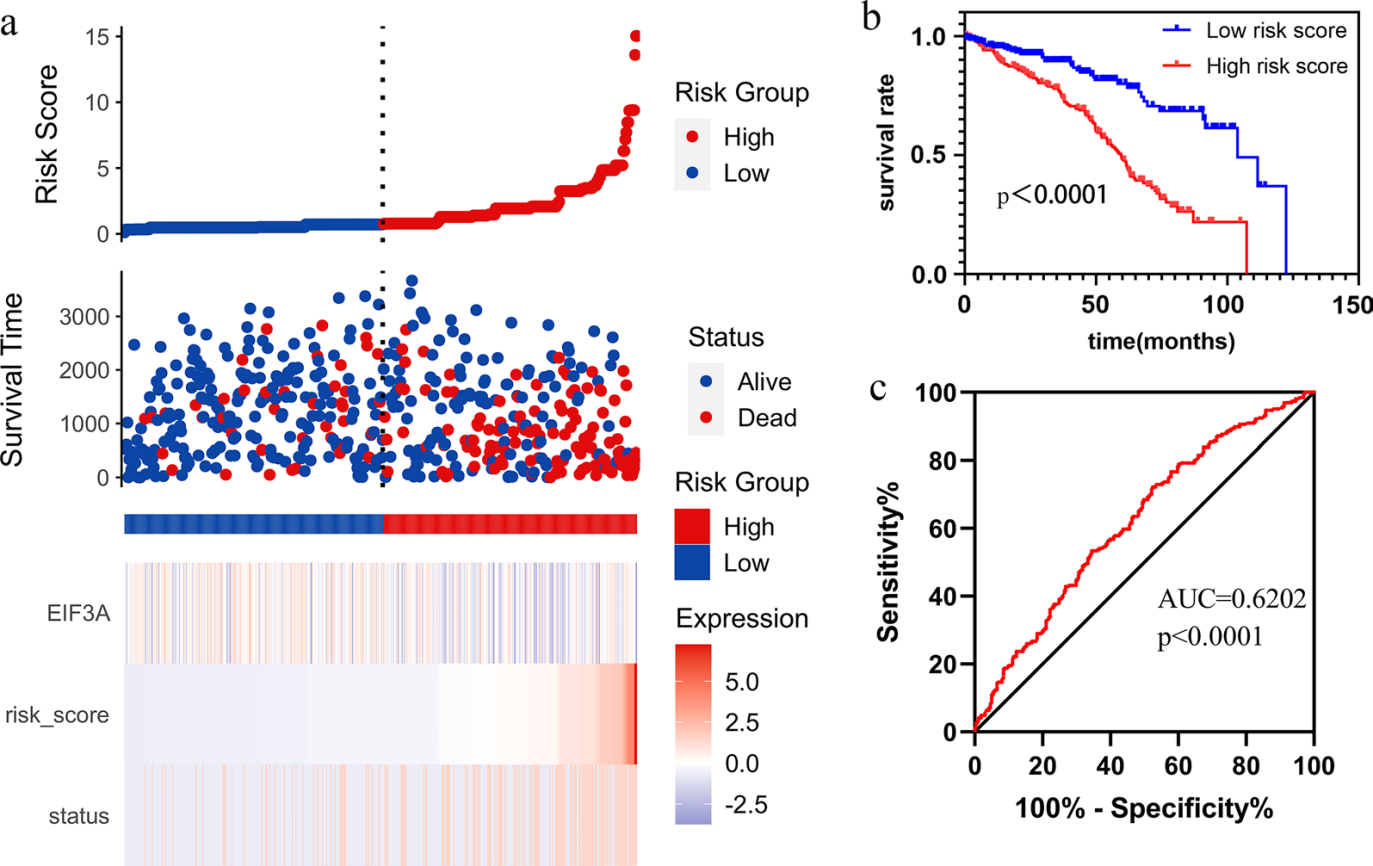
Calculation of the Risk score(RS) and validation of the prognostic risk signature; **a** The risk score distribution, survival status and prognostic risk gene expression; **b** The ROC curve to evaluate the predictive effect of prognostic characteristics; **c** The survival analysis showed that low-risk survival time was significantly more long than high-risk survival

Based on the results of multivariate analyses, multiple clinical prediction indicators were integrated and distributed in light of a certain proportion. To predict the 1-, 3-, and 5-year survival probabilities, the EIFA3A variables were combined with seven other clinicopathological parameters as shown in Fig. 4b. In addition, if we can predict tumour progression and prognosis in advance, perhaps treatments or decisions can be appropriately modified. The risk score (RS) was calculated via the results of multivariate Cox regression analysis (Table 3). Then, the ccRCC patients were divided into high-risk and low-risk groups by the median risk score calculated above. The results demonstrated that patients in the low-risk group had a much better survival than those in the high-risk group (Fig. 5a), which was also verified by the K-M curve (Fig. 5b). ROC curve analysis was used to evaluate the prediction efficiency of RS, which indicated that the AUC for the RS values was 0.6202 (Fig. 5c).

### Exploring the possible cellular mechanism by GSEA

**Fig. 6:**
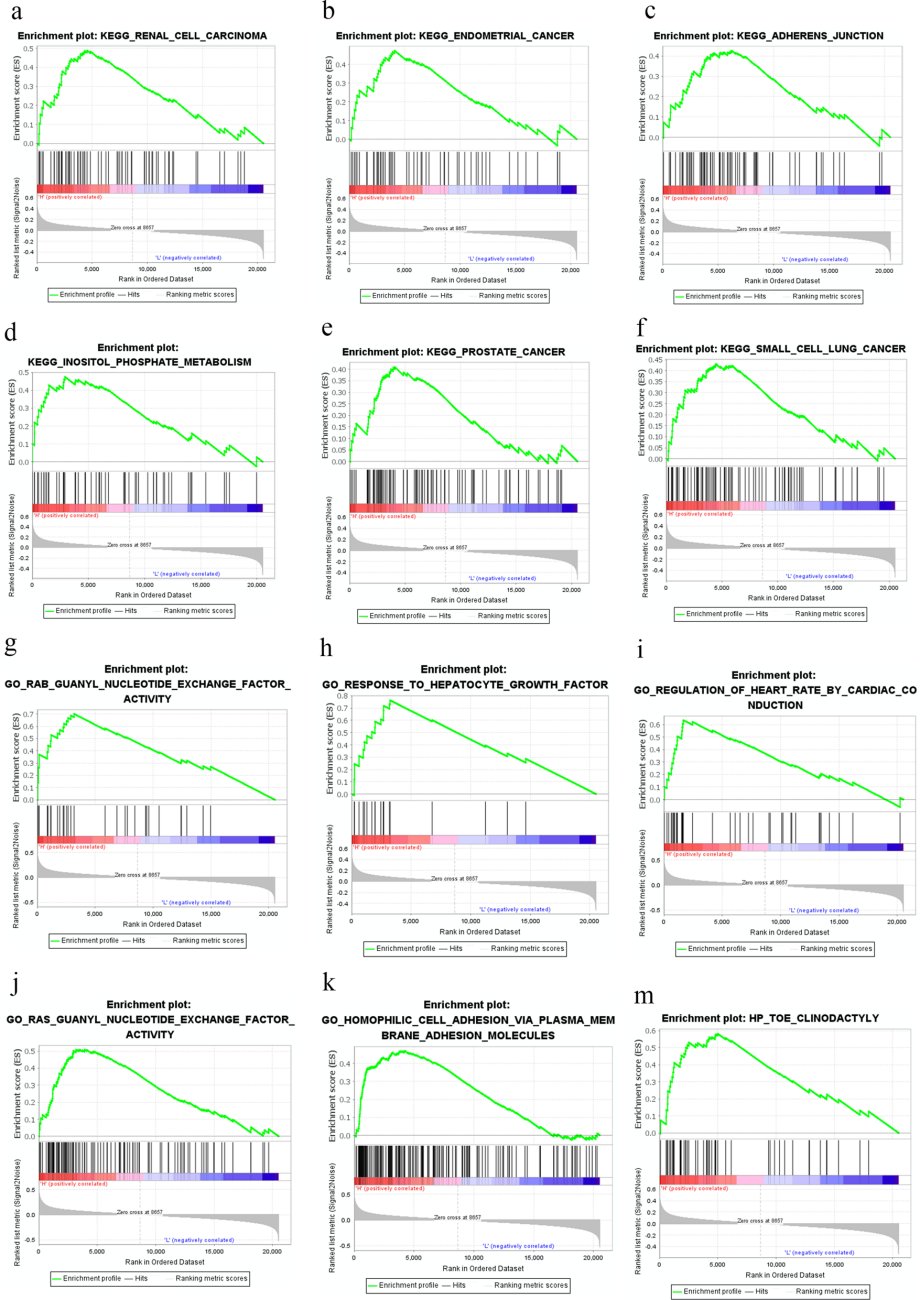
Enrichment plots from gene set enrichment analysis (GSEA) in KEGG database; **a** renal cell carcinoma pathway; **b** endometrial cancer pathway; **c** adherens junction pathway; **d** inositol phosphate metabolism pathway; **e** prostate cancer pathway; **f** small cell lung cancer pathway; Enrichment plots from gene set enrichment analysis (GSEA) in GO database; **g** Rab-guanine nucleotide exchange factor activity (Rab-GEFs); **h** response to hepatocyte growth factor pathway (HGF pathway); **i** regulation of heart rate by cardiac conduction pathway; **j** ras guanyl nucleotide exchange factor activity (Ras-GEFs); **k** homophilic cell adhesion via plasma membrane adhesion molecules pathway; **m** toe clinodactyly pathway

Based on the median EIF3A expression, we performed Gene Set Enrichment Analysis (GSEA) between tissues from the two groups. The results suggested that the related signalling pathways in KEGG (Additional file 1: Table S2), containing renal cell carcinoma (NES = 1.94, normalized p = 0.009, FDR q = 0.12), endometrial cancer (NES = 1.9, normalized p = 0.003, FDR q = 0.08), adherens junction (NES = 1.76, normalized p = 0.013, FDR q = 0.22), inositol phosphate metabolism (NES = 1.72, normalized p = 0.009, FDR q = 0.23), prostate cancer (NES = 1.70, normalized p = 0.01, FDR q = 0.23), small cell lung cancer (NES = 1.66, normalized p = 0.02, FDR q = 0.25) (Additional file 1: Table S2, Fig. 6). Additional file 1: Table S1 and Fig. 6 show that EIF3A expression was related to signalling pathways including rab guanyl nucleotide exchange factor activity (NES = 2.43, normalized p = 0.00, FDR q = 0.00), response to hepatocyte growth factor (NES = 2.41, normalized p = 0.00, FDR q = 0.00), regulation of heart rate by cardiac conduction (NES = 2.35, normalized p = 0.00, FDR q = 0.002), ras guanyl nucleotide exchange factor activity (NES = 2.34, normalized p = 0.00, FDR q = 0.002), homophilic cell adhesion via plasma membrane adhesion molecules (NES = 2.32, normalized p = 0.00, FDR q = 0.003), toe clinodactyly (NES = 2.27, normalized p = 0.00, FDR q = 0.008). These results provide clues regarding the underlying mechanism in the pathogenesis of ccRCC.

### Coexpression of EIF3A in clear cell renal cell carcinoma

**Fig. 7:**
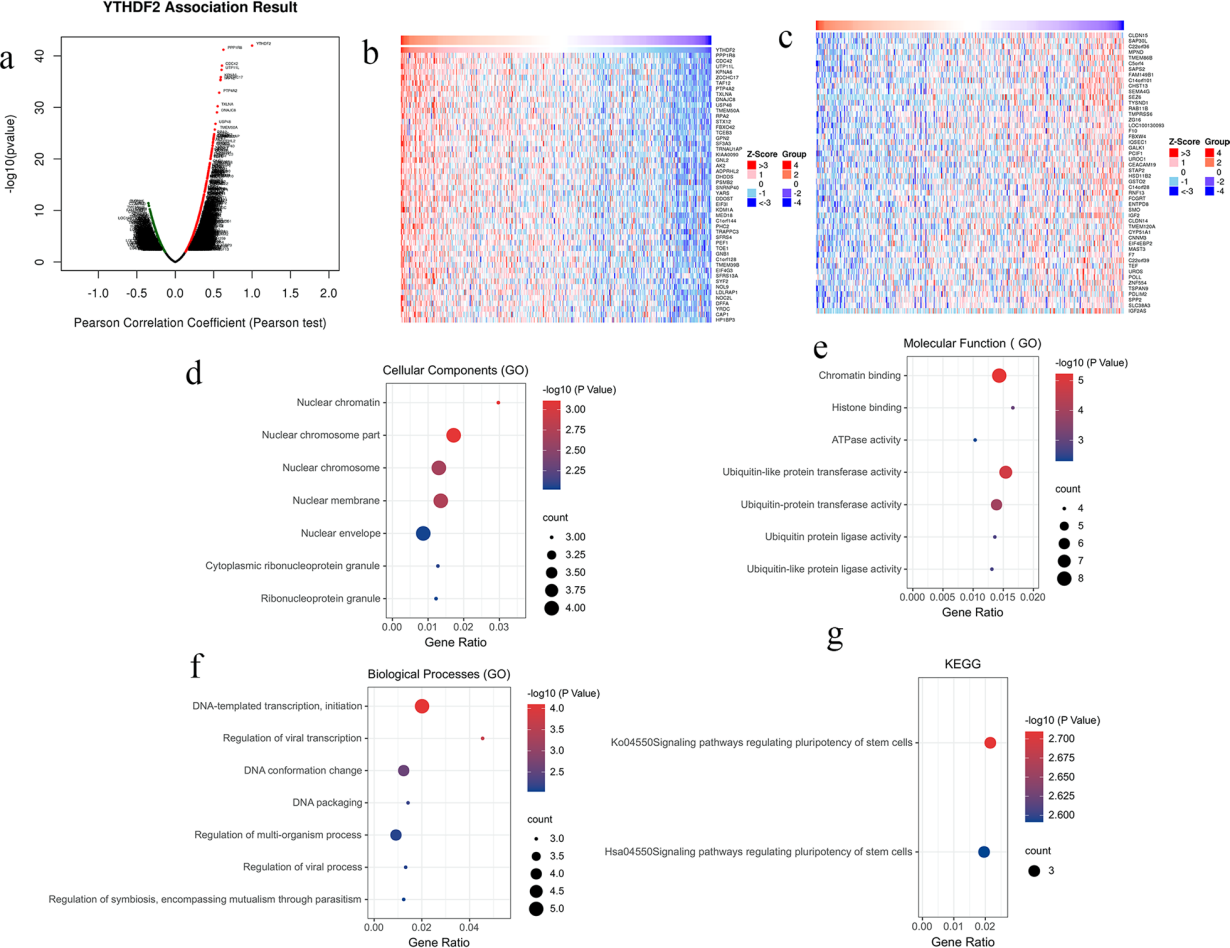
The analysis of EIF3A co-expression genes. **a** Volcano plot of all genes co-expressed with EIF3A; The top 50 genes positively (**b**) and negatively (**c**) correlated with EIF3A co-expression in ccRCC; The potential function analysis of EIF3A co-expression genes by using the GO database and the KEGG database, **d** Molecular Function, **e** Biological processes, **f** Cellular components, **g** KEGG database

To investigate the potential pathogenesis of EIF3A in ccRCC, we analysed the coexpression genes of EIF3A in renal clear cell carcinoma by using the LinkedOmics database. Based on the value of Pearson’s correlation ≥ 0.7 and FDR < 0.05, there were a total of 87 coexpressed genes with FIE3A expression in ccRCC. The EIF3A association results revealed that the total genes were pertinent to EIF3A by Pearson’s correlation coefficient. Figure 7a, b and c indicate the top 50 positively and negatively correlated genes in the form of heatmaps. Then, we analysed the pathway, function and intracellular localization of these coexpressed genes by using the GO and KEGG databases and presented the results in bubble plots. Figure 7d demonstrates that these genes were mainly distributed in the nuclear chromosome part, nuclear chromosome, nuclear membrane and nuclear envelope, and are mainly involved in chromatin binding, ubiquitin-like protein transferase activity, transcription factor binding, and ATPase activity (Fig. 7e). For participation in metabolism, most of them are involved in the regulation of viral transcription, DNA conformation change, DNA-templated transcription, and initiation (Fig. 7f). The results of the KEGG database revealed that these genes were mostly enriched in signalling pathways regulating the pluripotency of stem cells (Fig. 7g).

### Establish a PPI network of EIF3A-coexpressed genes and identify and analyse potential “Hub” genes

**Fig. 8:**
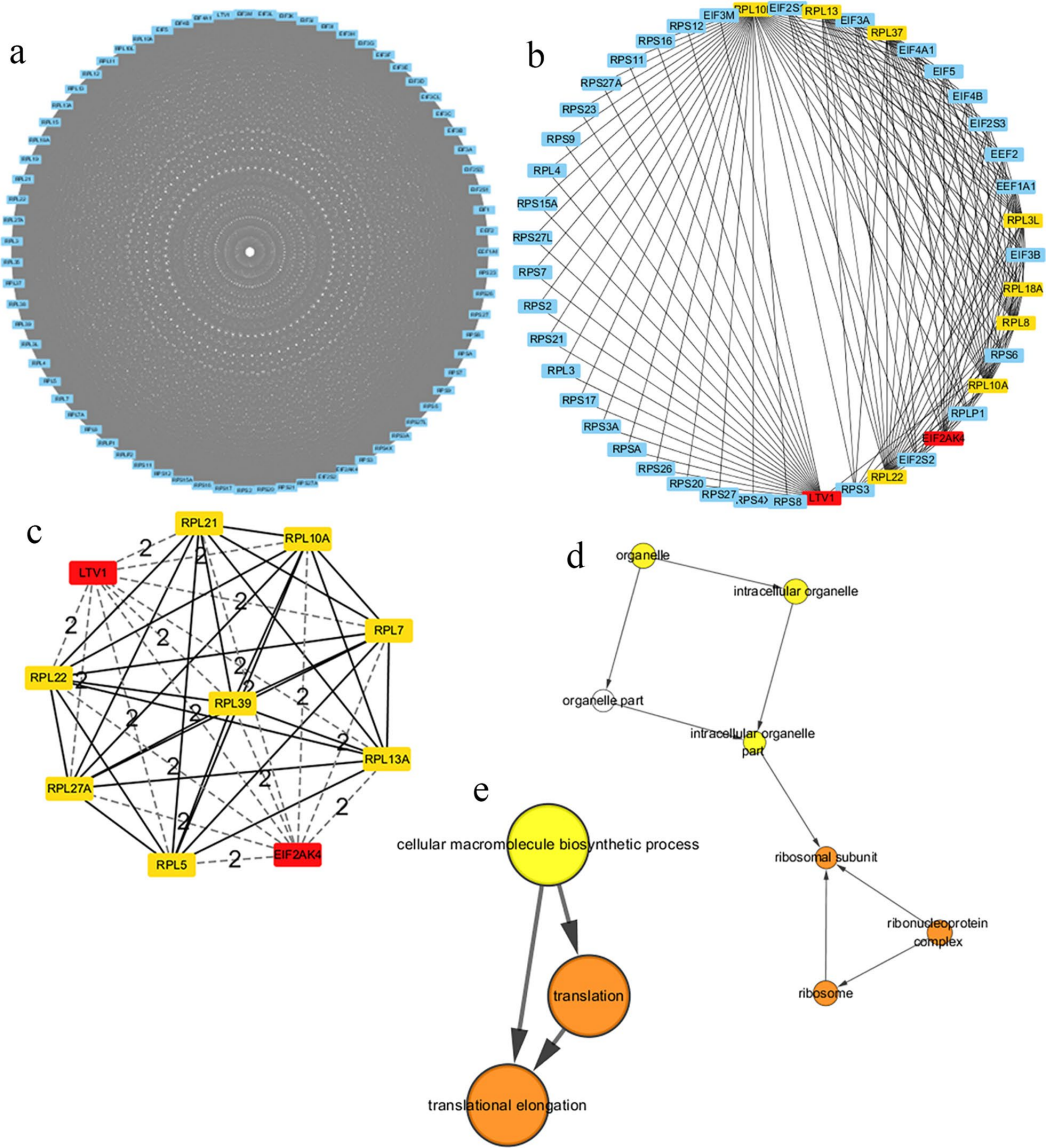
Establishment of the PPI network of EIF3A related genes and recognition hub gene. **a** The PPI network of EIFA co-expressed genes; **b** the top 10 genes in PPI network; **c** The interaction network of the top 10 genes; the analyses of biological processes of all hub genes that revealed (**d**) intracellular localization and (**e**) intracellular biological functions

The PPI network contained 70 coexpressed genes. It was constructed by using the STRING database and visualized in Cytoscape 3.7.2 (Fig. 8a). Then, by means of the CytoHubba plugin, the top ten genes were screened out on the basis of the degree score of each gene node (Fig. 8b, c). The results of Bingo plugin analysis revealed the biological process of the top ten genes (Fig. 8d, e). Given that, the hub genes were identified as LTV1 and EIF2AK4.

**Fig. 9:**
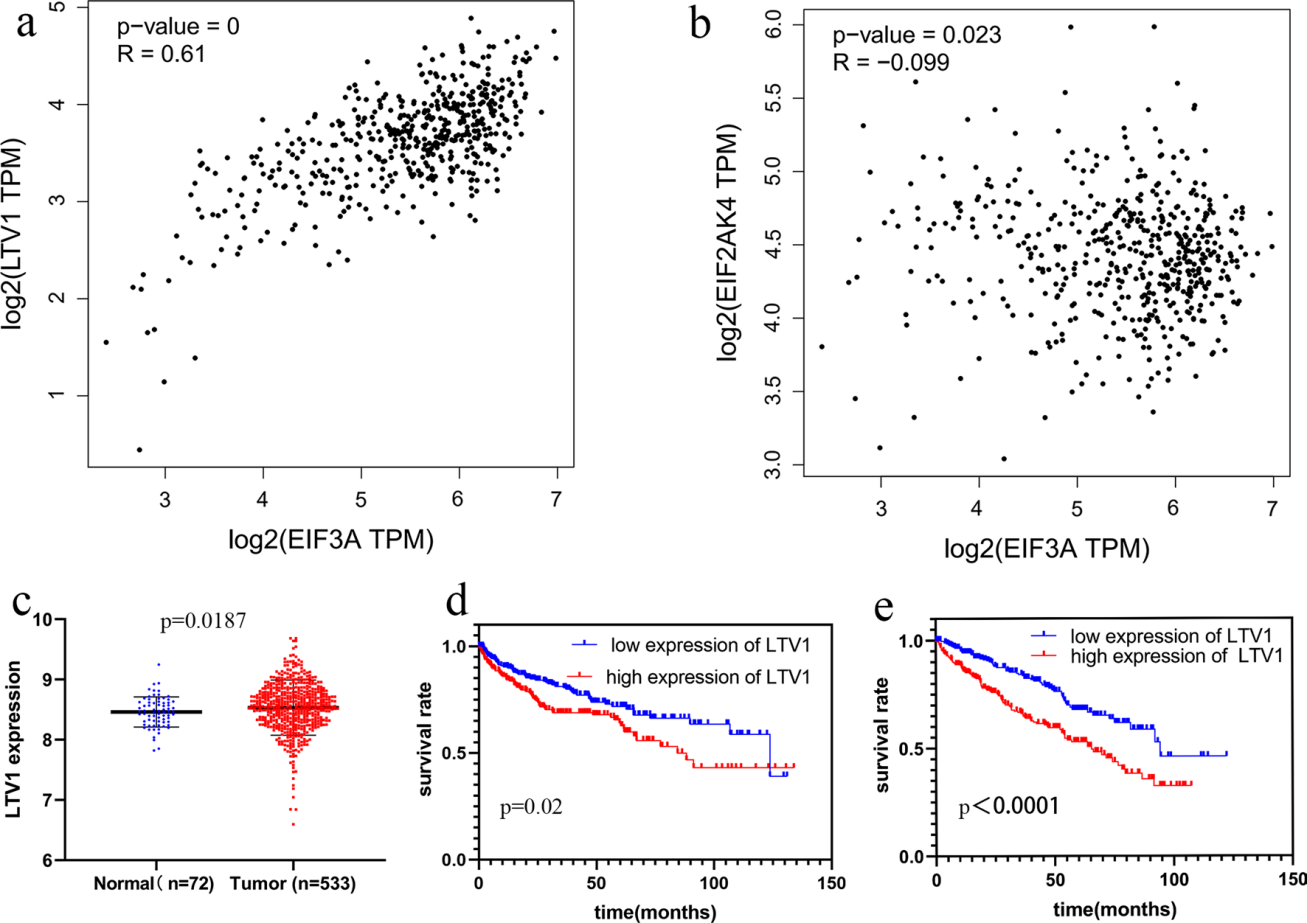
Identification of the hub genes and the expression level of LTV1 in ccRCC. **a**, **b** The analysis of correlation EIF3A with LTV1 and EIF2AK4; **c** The expression level of LTV1 in 72 ccRCC tissues and 72 normal tissues; **d** The expression level of LTV1 in 533 ccRCC tissues and 72 normal tissues; Kaplan–Meier survival curve shows LTV1 expression as **e** DFS and **f** OS in ccRCC

To investigate the correlation between the two genes and IEF3A, we used the GEPIA database to analyse the two hub genes in turn. The results showed that there was a good correlation between EIF3A and LTV1 (P < 0.001, R = 0.61) (Fig. 9a) but not EIF2AK4 (P = 0.023, R = − 0.099) (Fig. 9b). Furthermore, we identified proteins located mainly in the ribosome or ribosome subunit (Fig. 8d) that participated mainly in the cellular macromolecule biosynthetic process (Fig. 8e), suggesting that they may affect protein biosynthesis in cells.

The expression and prognostic value of LTV1 was verified in ccRCC, and the results confirmed that LTV1 had higher expression in tumour tissue (Fig. 9c, d). Kaplan–Meier analyses with log-rank tests indicated that the higher the expression of LTV1 was, the shorter the survival time for OS and DFS (Fig. 9e, f).

### EIF3A expression correlated with immune cell infiltration in renal clear cell carcinoma

**Fig. 10:**
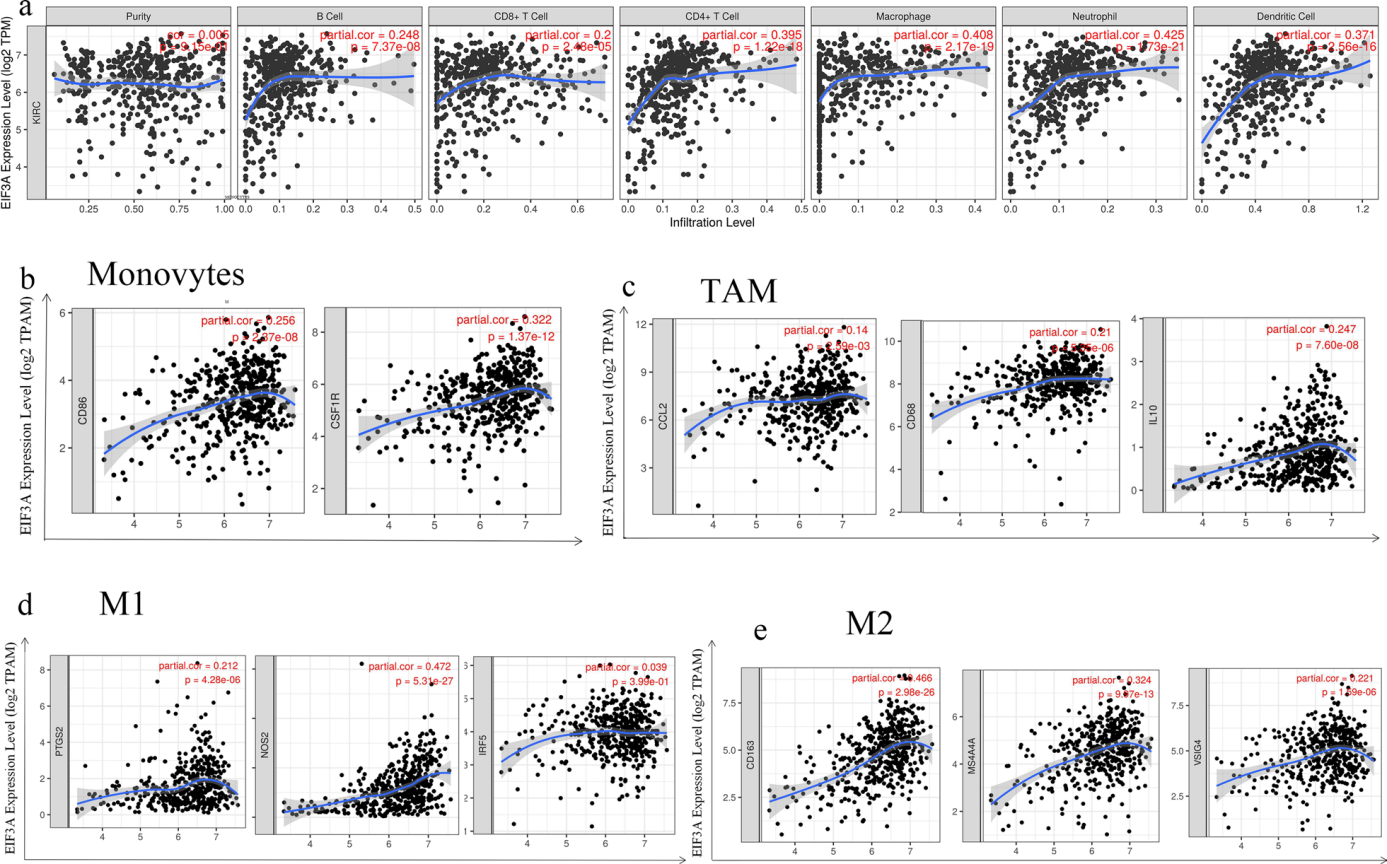
EIF3A expression is correlated with the infiltration of immune cells in ccRCC. **a** EIF3A expression is correlated with the infiltration of B cells, CD8 + T cells, CD4 + T cells, macrophages, neutrophils, and dendritic cells in ccRCC; **b**–**e** Scatterplots of correlations between EIF3A expression and gene markers of monocytes (**b**), TAMs (**c**), and M1 (**d**) and M2 macrophages (**e**) in ccRCC

Clear cell renal cell carcinoma (ccRCC) is a highly immune-infiltrated tumor [26, 27]. Postoperative recurrence of ccRCC is associated with lower T cell infiltration, lower adaptive immune response, low Teff/Treg ratio, and higher neutrophilic gene expression [28]. Furthermore, immune-related treatment is a feasible immunotherapy method for malignant tumours, and immunotherapy would help to achieve a better prognosis for inoperable patients [29]. In this context, we analysed the correlation between EIF3A and the level of ccRCC immune infiltration using the TIMER database (Fig. 10a). EIF3A expression was significantly correlated with B cells (r = 0.248, P = 7.37 × 10^–8^), CD8 + T cells (r = 0.2, P = 2.48 × 10^–5^), CD4 + T cells (r = 0.395, P = 1.22 × 10^–18^), macrophages (r = 0.408, P = 2.17 × 10^–21^), neutrophils (r = 0.425, P = 1.73 × 10^–21^), and DCs (r = 0.371, P = 2.56 × 10^–16^). In increase in EIF3A expression was associated with a general increase in the immune infiltration level, especially in the macrophages and neutrophils.

### EIF3A expression correlated with immune marker genes

Next, to further investigate the role of EIF3A in immunity, we explored the relationship between EIF3A expression and markers of different immune cell types in renal clear cell carcinoma. The results showed that EIF3A in ccRCC was positively correlated with Monocyte, TAM, M1 and M2 Macrophage (except IRF5), Neutrophil, Dendritic cell, Th1 (except IFN-y), Th2 (except GATA3 and IL13), BCL6 in Tfh, STAT3 in Th17, Treg (except FOXP3) and TIM-3 in T cell exhaustion (Additional file 1: Table S3) (Fig. 10b–e). We noticed that the expression of EIF3A and related immune cell markers was more significant in regulatory T cells and Myeloid-derived suppressor cells. Subsequently, we conducted immunofluorescence experiments. The results showed that EIF3A expression was meaningless in regulatory T cells (Foxp3) and in ccRCC, but showed a relatively high expression in Myeloid-derived suppressor cells (MDSC) cells (CD11b) (Additional file 1: Fig. S2). Similarly, negatively related gene markers were mostly concentrated in T cell exhaustion (PD-1, LAG3 and GZMB) and IL13 in Th2 cells (Additional file 1: Table S3). These correlations remained nearly unchanged after tumour purity. Among these gene marks, STAT5B, STAT3 and BDCA-4 had a superior correlation with EIF3A expression. The expression relationships of YTHDF2 with monocytes and TAMS markers were consistent with the TIMER database (Table 4). Above all, the relationship of EIF3A and the infiltration of immune cells of different phenotypes can affect the occurrence and development of renal carcinoma.

**Table 4:** The correlation of analysis between EIF3A and relate genes and markers of monocyte, TAM and macrophages in GEPIA dataset

Description	Gene markers	GEPIA			
		Tumor		Normal	
		R	p-value	R	p-value
Monocyte	CD86	0.29	**1.5e − 11**	0.21	0.071
	CD115 (CSF1R)	0.35	**0**	0.2	**0.086**
TAM	CCL2	0.1	**0.019**	0.35	**0.0023**
	CD68	0.35	**4.4e − 16**	− 0.01	0.93
	IL10	0.26	**1.9e − 09**	0.25	**0.031**
M1 Macrophage	INOS (NOS2)	0.23	**6.8e − 08**	0.29	**0.012**
	IRF5	0.096	**0.028**	0.29	**0.013**
	COX2 (PTGS2)	0.051	0.24	0.34	**0.0032**
M2 Macrophage	CD163	0.24	**1.5e − 08**	0.18	0.14
	VSIG4	0.22	**5.7e − 07**	0.15	0.2
	MS4A4A	0.34	**2.2e − 15**	0.14	0.24

*TAM* tumour-correlated macrophage

R-value of Spearman’s correlation

Bold font: p value < 0.05

## Discussion

Recently, an increasing number of studies have focused on m6A interactions in cancer [30]. A potential role of m6A methylation in tumorigenesis and progression has been well documented [31]. Clear cell renal cell carcinoma (ccRCC) is the most common histological type of RCC, and several M6a-related genes have been shown to be associated with OS and/or DFS in ccRCC [32–34]. EIF3A is a highly conserved gene that may also be involved in the regulation of cellular, physiological, and pathological processes, not only in cancer [35]. However, EIF3A, as a “reader”, has hardly been mentioned in ccRCC. The expression of EIF3A is different from that of other genes, being expressed at a low level in normal tissues, increases significantly in the presence of cancer, and decreases again in high-grade tumours [35]. EIF3A may be essential for the maintenance of the malignant status of cells and thus affects the prognosis [21]. Hence, we systematically investigated the prognostic significance of EIF3A in ccRCC.

In this study, we investigated the relationship between EIF3A expression, clinicopathological parameters and patient survival outcomes based on the TCGA database and ccRCC tissue array. The results revealed that the correlation between EIF3A and ZC3H13 was the highest among 29 m6A-related genes; 16 genes were upregulated and 13 genes were downregulated in ccRCC. These discrepant EIF3A expression levels in different cancers are the result of different underlying mechanisms with distinct biological properties, and it has been demonstrated that high expression of EIF3A is associated with cell proliferation, colony formation, wound healing, migration and invasion in lung, urinary bladder and pancreatic cancer cells [17, 19, 20]. In clear cell renal cell carcinoma, EIF3A expression is lower in the tumour tissue. Additionally, high EIF3A expression was significantly associated with better pathologic stage, histological grade, T stage, and M stage. At the protein expression level, the IHC results revealed that EIF3A staining was weaker in ccRCC tissues than in normal tissues (Fig. 2d). Moreover, the overall survival was related to the clinical stage, grade, M stage, age and EIF3A expression (Table 3). Univariate and multivariate Cox regression analyses demonstrated that EIF3A expression was associated with a poor prognosis in patients with renal cancer. Interestingly, EIF3A was relatively downregulated in ccRCC and negatively correlated with the degree of malignancy of the tumour. For patient prognosis, analysis of EIF3A in Kaplan–Meier analyses with log-rank tests indicated that decreased EIF3A expression was related to an unfavourable prognosis in ccRCC (OS and DFS). Therefore, survival analysis of our own microarray samples was performed. Furthermore, the AUC for RS was 0.6202, a convincing prognostic value for overall survival of ccRCC patients.

Overall, DNA synthesis decreased by approximately 50% when antisense cDNA was used to reduce EIF3A expression [20]. In another study, inhibition of EIF3A expression increased epidermal growth factor (EGF) stimulation of DNA synthesis [36]. Multiple studies have shown that low EIF3A subunit expression reduces ribonucleotide-reductase M2 [20] expression and stimulates p27kip1 synthesis [20] and N-myc downstream regulated gene-1 (NDRG1) [37]. The conclusions of the above studies suggest that YTHDF2 plays a dual and complex role in tumour cells. Therefore, GSEA was also conducted to explore how EIF3A participates in ccRCC pathogenesis, and the results revealed that the pathways with strong correlations included renal cell carcinoma, endometrial cancer, adherens junctions, rab guanyl nucleotide exchange factor activity, response to hepatocyte growth factor, and response to hepatocyte growth factor.

The results of coexpression analyses revealed that there was a strong positive correlation between EIF3A expression and LTV1 expression. LTV1 is one of many assembly factors (AFs) and it is involved in assembling the small (40S) ribosomal subunit [38, 39]. Because of the increasing demand for protein synthesis, the ribosomal assembly pathway is upregulated in all cancers [40, 41]. In one study, it was shown that LTV1 was substoichiometric in breast cancer cells, producing reduced RPS10 and RACK1 ribosomes [42]. Furthermore, knockdown of LTV1 attenuated SR-3029-induced apoptosis in MDA-MB-231 breast cancer cells [43] and restored drug sensitivity [43, 44]. Therefore, we compared the differential LTV1 expression in ccRCC tissues and paracancerous tissues, finding that LTV1 expression was significantly higher than that in nontumour tissues. In view of the results of the survival analysis, high expression of LTV1 was associated with poor survival outcomes. We hypothesized that LTV1 and EIF3A could jointly promote the tumorigenesis of clear cell renal cell carcinoma and significantly affect the prognosis.

Another important finding of this study is that EIF3A is associated with the degree of immune invasion in various tissues. The expression of EIF3A was correlated with various immune cells to different degrees, among which its expression was moderately positively correlated with macrophages and neutrophils and weakly positively correlated with the B cells, CD8 + , CD4 + , DCs and neutrophils. We found that the correlation between EIF3A and M1/M2 macrophage markers, including PTGS2, IRF5, CD163, VSIG4 and MS4A4A, and markers of M1 macrophages was stronger than that of markers of M2 macrophages. Moreover, EIF3A was related to TAM markers, which suggested a potential regulatory role of YTHDF2 in TAM polarization. This study also found that the two most closely positively related markers were STAT3 (markers of Th17) and STAT5B (markers of Tregs), which indicated that EIF3A could activate and stimulate Tregs and Th17 cells. In addition to these two T cells, there are multiple markers of other T cells associated with EIF3A expression, including Th1, Th2 and Tfh cells. Moreover, Tim-3, a key gene in T cell exhaustion, was positively correlated with the expression of EIF3A, but negative correlations were found for T cell exhaustion markers, including PD-1, LAG-3 and GZMB, which demonstrated that the potency of EIF3A to induce infiltration of T cell exhaustion may not unidirectionally promote or suppress T cell-mediated immunity. Therefore, it is reasonable to surmise that EIF3A has an important role in regulating immune cell recruitment and activation in ccRCC.

Taken together, we identified for the first time genetic alterations in EIF3A in ccRCC and found a clear relationship between alterations leading to an increase in EIF3A levels and worse clinical characteristics, including survival. EIF3A is a crucial regulator of m6A modifications, tumorigenesis and progression. The results of this study may provide a potential direction and new insights into the pathogenesis of M6A-related genes in ccRCC, which are conducive to the development of new targeted drugs. Our results call for further experimental studies for validation and to clarify the mechanism by which EIF3A affects ccRCC.

## Supplementary Information

**Additional file 1:** **Table S1.** Gene sets enrichment analysis of high EIF3A mRNA expression level in the ccRCC based on KEGG database. **Table S2.** Gene sets enrichment analysis of high EIF3A mRNA expression level in the ccRCC based on GO database. **Table S3.** The correlation of analysis between EIF3A and relate genes markers of immune cells in TIMER datase. **Figure S1.** The expression level of EIF3A in colon para-cancerous tissues and pancreatic para-cancerous tissues; Immunohistochemical (IHC) analysis of EIF3A expression in colon para-cancerous tissues (a) and pancreatic para-cancerous tissues(b). **Figure S2.** Eif3A was expressed in Myeloid-derived suppressor cells and Regulatory T cells by immunofluorescence assay; a Myeloid-derived suppressor cells (MDSC) cells (white arrow) were double immunostained with anti-CD11b antibody (red) and anti-EIF3A antibody (green). The cell nuclei were counterstained with DAPI (blue). The co-localization between the two endogenous proteins CD11b and EIF3A is shown in the merge panel. Scale bar, 20 mm; b Regulatory T cells (white arrow) were double immunostained with anti-Foxp3 antibody (red) and anti-EIF3A antibody (green). The cell nuclei were counterstained with DAPI (blue). The co-localization between the two endogenous proteins Foxp3 and EIF3A is shown in the merge panel. Scale bar, 20 mm.

## Data Availability

The RNA-sequencing data and corresponding clinical information were downloaded from The Cancer Genome Atlas (TCGA) database.
