# Supplementary material for: Systematic analyses of the role of prognostic and immunological EIF3A, a reader protein, in clear cell renal cell carcinoma
Source: Cancer Cell Int. 2021 Dec 19;21:680. doi: 10.1186/s12935-021-02364-2 (PMC8684683; doi:10.1186/s12935-021-02364-2)

Supplementary Table.1 Gene sets enrichment analysis of  high EIF3A mRNA expression level in the ccRCC based on KEGG database

Supplementary Table.2 Gene sets enrichment analysis of  high EIF3A mRNA expression level in the ccRCC based on GO database

| NAME | NES | NOM p-val | FDR q-val |
| --- | --- | --- | --- |
| GO RAB GUANYL NUCLEOTIDE EXCHANGE FACTOR ACTIVITY | 2.438 | 0.000 | 0.000 |
| GO RESPONSE TO HEPATOCYTE GROWTH FACTOR | 2.416 | 0.000 | 0.000 |
| GO REGULATION OF HEART RATE BY CARDIAC CONDUCTION | 2.351 | 0.000 | 0.003 |
| GO RAS GUANYL NUCLEOTIDE EXCHANGE FACTOR ACTIVITY | 2.347 | 0.000 | 0.003 |
| GO HOMOPHILIC CELL ADHESION VIA PLASMA MEMBRANE ADHESION MOLECULES | 2.324 | 0.000 | 0.003 |
| HP TOE CLINODACTYLY | 2.271 | 0.000 | 0.009 |

| NAME | NES | NOM p-val | FDR q-val |
| --- | --- | --- | --- |
| KEGG RENAL CELL CARCINOMA | 1.944 | 0.010 | 0.127 |
| KEGG ENDOMETRIAL CANCER | 1.909 | 0.004 | 0.088 |
| KEGG ADHERENS JUNCTION | 1.764 | 0.014 | 0.226 |
| KEGG INOSITOL PHOSPHATE METABOLISM | 1.729 | 0.010 | 0.236 |
| KEGG PROSTATE CANCER | 1.704 | 0.012 | 0.232 |
| KEGG SMALL CELL LUNG CANCER | 1.669 | 0.025 | 0.251 |

Supplementary Table.3 The correlation of analysis between EIF3A and relate genes markers of immune cells in TIMER datase

| Description | Gene markers | KIRC | | | |
| --- | --- | --- | --- | --- | --- |
|  |  | None | | Purity | |
|  |  | Cor | P | Cor | P |
| CD8+ T cell | CD8A | 0.005741529 | 0.894791793 | 0.002362323 | 0.959657336 |
|  | CD8B | -0.050985313 | 0.239959431 | -0.061465399 | 0.187710927 |
| T cell (general) | CD3D | -0.087934319 | **0.042429013** | -0.092934551 | **0.046120377** |
|  | CD3E | -0.026929901 | 0.535009016 | -0.031318041 | 0.502369702 |
|  | CD2 | -0.002720506 | 0.950036921 | -0.001538838 | 0.973714008 |
| B cell | CD19 | -0.049847656 | 0.250624044 | -0.045258097 | 0.332250248 |
|  | CD79A | -0.057717629 | 0.183354948 | -0.068971982 | 0.139238672 |
| Monocyte | CD86 | 0.242904925 | **1.35E-08** | 0.256347938 | **2.37E-08** |
|  | CD115 (CSF1R) | 0.321436741 | **2.82E-14** | 0.322120959 | **1.37E-12** |
| TAM | CCL2 | 0.087834386 | **0.042665237** | 0.140013892 | **0.002587484** |
|  | CD68 | 0.238860299 | **2.37E-08** | 0.210132224 | **5.35E-06** |
|  | IL10 | 0.246483408 | **8.10E-09** | 0.247175922 | **7.60E-08** |
| Ml Macrophage | INOS (NOS2) | 0.48693774 | **4.40E-33** | 0.472389607 | **5.31E-27** |
|  | IRF5 | 0.043050768 | 0.321182553 | 0.039343203 | 0.399358567 |
|  | COX2(PTGS2) | 0.178456126 | **3.42E-05** | 0.212260069 | **4.28E-06** |
| M2 Macrophage | CD163 | 0.477960727 | **8.97E-32** | 0.466193785 | **2.98E-26** |
|  | VSIG4 | 0.24869603 | **5.89E-09** | 0.221354942 | **1.59E-06** |
|  | MS4A4A | 0.323879593 | **1.75E-14** | 0.323998032 | **9.97E-13** |
| Neutrophils | CD66b(CEACAM8) | 0.097462204 | **0.024438391** | 0.085582716 | 0.066369532 |
|  | CD11b (ITGAM) | 0.341755182 | **4.77E-16** | 0.335347791 | **1.40E-13** |
|  | CCR7 | 0.171516771 | **6.89E-05** | 0.167474525 | **0.000304305** |
| Natural killer cell | KIR2DL1 | 0.092474487 | **0.032801672** | 0.056076272 | 0.229485364 |
|  | KIR2DL3 | 0.07885037 | 0.068917351 | 0.06085747 | 0.19212209 |
|  | KIR2DL4 | -0.080394079 | 0.063640956 | -0.0970121 | **0.037324511** |
|  | KIR3DL1 | 0.118688467 | **0.006080546** | 0.102029973 | **0.028491752** |
|  | KIR3DL2 | -0.006984636 | 0.872190896 | -0.01607433 | 0.730684041 |
|  | KIR3DL3 | 0.016907167 | 0.696947958 | 0.030037927 | 0.52000618 |
|  | KIR2DS4 | 0.041245775 | 0.341908274 | 0.018541623 | 0.691324061 |
| Dendritic cell | HLA-DPB1 | 0.207502008 | **1.35E-06** | 0.200860215 | **1.39E-05** |
|  | HLA-DQB1 | 0.142114692 | **0.001001935** | 0.137011985 | **0.003201493** |
|  | HLA-DRA | 0.142114692 | **0.001001935** | 0.24745097 | **7.35E-08** |
|  | HLA-DPA1 | 0.240923661 | **1.78E-08** | 0.245990526 | **8.81E-08** |
|  | BDCA-1(CD1C) | 0.267865508 | **3.28E-10** | 0.270359047 | **3.65E-09** |
|  | BDCA-4(NRP1) | 0.608322721 | **2.94E-55** | 0.598701677 | **3.57E-46** |
|  | CD11c (ITGAX) | 0.118338833 | **0.006232969** | 0.133096794 | **0.004200514** |
| Th1 | T-bet (TBX21) | 0.115580485 | **0.007560874** | 0.121180374 | **0.009203704** |
|  | STAT4 | 0.101180067 | **0.019468439** | 0.114101329 | **0.014237351** |
|  | STAT1 | 0.4045272 | **2.11E-22** | 0.40852194 | **5.70E-20** |
|  | IFN-y (IFNG) | -0.076570954 | 0.077358751 | -0.088104112 | 0.058728951 |
|  | TNF-a (TNF) | 0.177262767 | **3.86E-05** | 0.186262198 | **5.73E-05** |
| Th2 | GATA3 | -0.066932701 | 0.122742337 | -0.021650119 | 0.642905613 |
|  | STAT6 | 0.485380352 | **7.47E-33** | 0.466336966 | **2.87E-26** |
|  | STAT5A | 0.311864567 | **1.74E-13** | 0.325539598 | **7.68E-13** |
|  | IL13 | -0.086231679 | **0.046607828** | -0.04229611 | 0.364895614 |
| Tfh | BCL6 | 0.260838322 | **9.72E-10** | 0.266566689 | **6.12E-09** |
|  | IL21 | 0.049464841 | 0.254285727 | 0.042307892 | 0.364761927 |
| Th17 | STAT3 | 0.716453027 | **4.47E-85** | 0.71441248 | **3.45E-73** |
|  | IL17A | -0.027366803 | 0.528402527 | -0.001963837 | 0.96645809 |
| Treg | FOXP3 | -0.062141824 | 0.151951569 | -0.051319206 | 0.271505001 |
|  | CCR8 | 0.174544794 | **5.09E-05** | 0.197396413 | **1.96E-05** |
|  | STAT5B | 0.709882262 | **7.13E-83** | 0.698098064 | **1.35E-68** |
|  | TGF& (TGFB1) | 0.200366945 | **3.13E-06** | 0.177466028 | **0.000127904** |
| T cell exhaustion | PD-1 (PDCD1) | -0.092814323 | **0.032162869** | -0.093217239 | **0.045458958** |
|  | CTLA4 | -0.016294279 | 0.707419245 | 0.010141168 | 0.828087893 |
|  | LAG3 | -0.135432421 | **0.001725529** | -0.146488672 | **0.001611952** |
|  | TIM-3 (HAVCR2) | 0.220994973 | **2.55E-07** | 0.185963645 | **5.89E-05** |
|  | GZMB | -0.086568955 | **0.045753662** | -0.101884598 | **0.028719845** |

None, correlation without adjustment. Purity, tumor purity-correlated adjustments.TAM, tumour-correlated macrophage; Tfh, follicular helper T cell; Th, T helper cell; Treg, regulatory T cell.Cor, R-value of Spearman’s correlation.Bold font: p value < 0.05.

Figure.S1 The expression level of EIF3A in colon para-cancerous tissues and pancreatic para-cancerous tissues; Immunohistochemical (IHC) analysis of EIF3A expression in colon para-cancerous tissues(a) and pancreatic para-cancerous tissues(b)


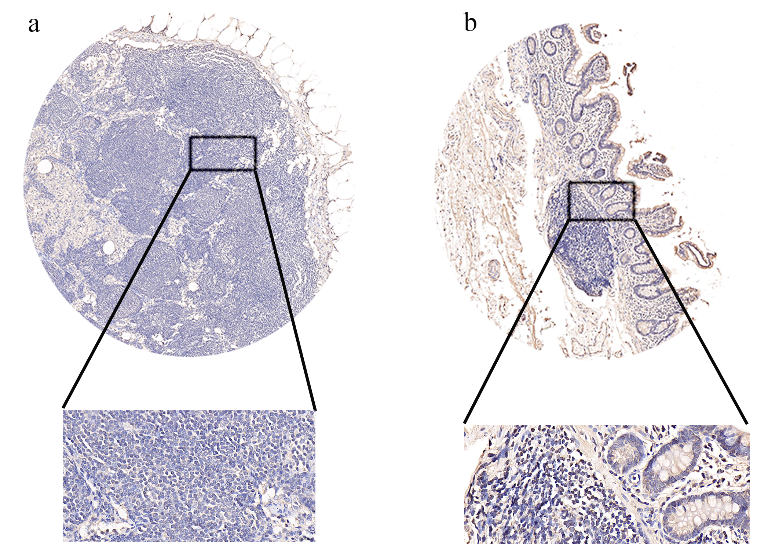


Figure.S2 Eif3A was expressed in Myeloid-derived suppressor cells and Regulatory T cells by immunofluorescence assay; a Myeloid-derived suppressor cells (MDSC)cells (white arrow) were double immunostained with anti-CD11b antibody (red) and anti-EIF3A antibody (green). The cell nuclei were counterstained with DAPI (blue). The co-localization between the two endogenous proteins CD11b and EIF3A is shown in the merge panel. Scale bar, 20 mm; b Regulatory T cells (white arrow) were double immunostained with anti-Foxp3 antibody (red) and anti-EIF3A antibody (green). The cell nuclei were counterstained with DAPI (blue). The co-localization between the two endogenous proteins Foxp3 and EIF3A is shown in the merge panel. Scale bar, 20 mm


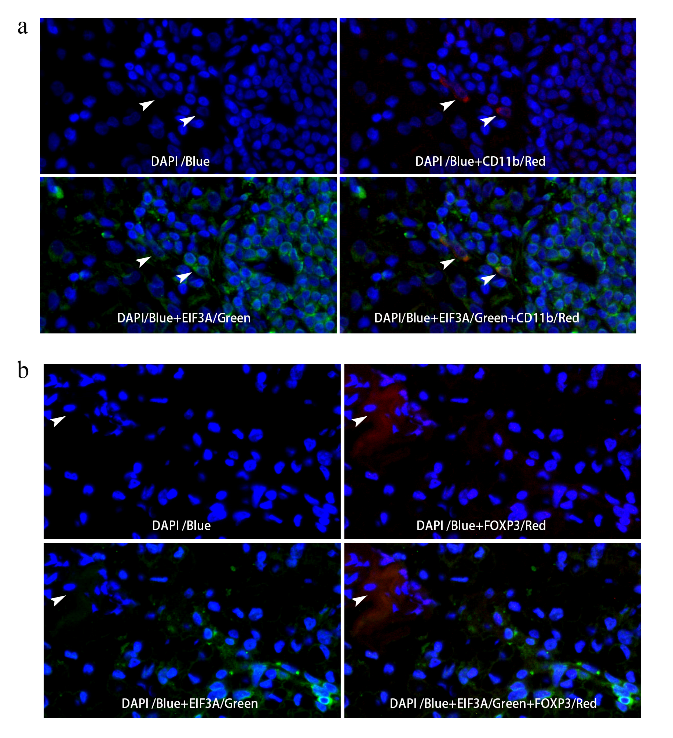

Supplement: Supplementary file 1 — Additional file 1: Table S1. Gene sets enrichment analysis of high EIF3A mRNA expression level in the ccRCC based on KEGG database. Table S2. Gene sets enrichment analysis of high EIF3A mRNA expression level in the ccRCC based on GO database. Table S3. The correlation of analysis between EIF3A and relate genes markers of immune cells in TIMER datase. Figure S1. The expression level of EIF3A in colon para-cancerous tissues and pancreatic para-cancerous tissues; Immunohistochemical (IHC) analysis of EIF3A expression in colon para-cancerous tissues (a) and pancreatic para-cancerous tissues(b). Figure S2. Eif3A was expressed in Myeloid-derived suppressor cells and Regulatory T cells by immunofluorescence assay; a Myeloid-derived suppressor cells (MDSC) cells (white arrow) were double immunostained with anti-CD11b antibody (red) and anti-EIF3A antibody (green). The cell nuclei were counterstained with DAPI (blue). The co-localization between the two endogenous proteins CD11b and EIF3A is shown in the merge panel. Scale bar, 20 mm; b Regulatory T cells (white arrow) were double immunostained with anti-Foxp3 antibody (red) and anti-EIF3A antibody (green). The cell nuclei were counterstained with DAPI (blue). The co-localization between the two endogenous proteins Foxp3 and EIF3A is shown in the merge panel. Scale bar, 20 mm. [file 12935_2021_2364_MOESM1_ESM.docx]
